# Processing Adipose Tissue Samples in a GMP Environment Standardizes the Use of SVF in Cell Therapy Treatments: Data on 302 Patients

**DOI:** 10.3390/biomedicines11092533

**Published:** 2023-09-14

**Authors:** Martina Cremona, Giulio Rusconi, Alessandro Ferrario, Luca Mariotta, Mauro Gola, Gianni Soldati

**Affiliations:** 1 Swiss Stem Cell Foundation, 6900 Lugano, Switzerland; martina.cremona@sscf.ch (M.C.);; 2 Swiss Stem Cells Biotech AG, 8008 Zürich, Switzerland

**Keywords:** adipose-derived stem cells, human cell therapy, mesenchymal stem cells, endothelial cells, pericytes, GMP-compliant-facility, clean room, ATMPs

## Abstract

Stromal vascular fraction (SVF) cells, together with adipose-derived mesenchymal stem cells, are becoming the tool of choice for many clinical applications. Currently, nearly 200 clinical trials are running worldwide to prove the efficacy of this cell type in treating many diseases and pathological conditions. To reach the goals of cell therapies and produce ATMPs as drugs for regenerative medicine, it is necessary to properly standardize GMP processes and, thus, collection methods, transportation strategies, extraction protocols, and characterization procedures, without forgetting that all the tissues of the human body are characterized by a wide inter-individual variability which is genetically determined and acquired during life. Here, we compare 302 samples processed under GMP rules to exclude the influence of the operator and of the anatomical site of collection. The influence of variability in the ages and genders of patients, along with laboratory parameters such as total cell number, cell viability, stem cell number, and other stromal vascular fraction cell subpopulations, has been compared. The results show that when the laboratory protocol is standardized, the variability of quantifiable cell parameters is widely statistically non-significant, meaning that we can take a further step toward standardized advanced cell therapy products.

## 1. Introduction

The importance and the role of adipose tissue has been largely re-evaluated after the discovery, in 2006, that it is the largest endocrine organ, interacting with all major organs through the production of a wide range of hormones and cytokines [1]. Adipose tissue is composed of different cell populations, such as mature adipocytes and their progenitors, endothelial cells (ECs) and endothelial progenitor cells (EPCs), constituting the vascular system of the tissue, immune cells, and mesenchymal stem cells (MSCs) [2,3]. MSCs were first isolated by Friedenstein and colleagues from the bone marrow and were later defined as multipotent stem cells with both self-renewal and differentiation abilities [4,5,6]. The first isolated bone-marrow-derived MSCs, when cultured, showed an intrinsic ability to form colonies with great cellular heterogeneity in terms of progenitors and undifferentiated stem cells, able to regenerate bone rudiments. Thanks to their multipotency, it was proven that they were able to differentiate three different cell types: osteoblasts, chondrocytes, and adipocytes [4]. MSCs are found in many tissues, including bone marrow, the umbilical cord, placental tissue, and adipose tissue. The stem cells in adipose tissue were identified as adipose-derived stem cells (ASCs), first isolated from adipose tissue by Zuk and colleagues in 2001 [2]. ASCs are plastic-adherent, multipotent, proliferative stem cells which can remain undifferentiated, self-renew, or undergo multilineage differentiation [7,8]. Since their first isolation more than 20 years ago, they have raised increasing interest in the field of regenerative and aesthetic medicine thanks to their regenerative potential, outlined by several studies which showed that these cells can differentiate along multiple pathways [9].

Historically, the first use of ASCs was in reconstructive surgery: autologous fat transplant was performed for aesthetic purposes, but due to the long-term problems of volume maintenance, additional transplants were required over time. Currently, to overcome this problem, SVF cells are cryopreserved and thawed secondarily to re-treat patients. This procedure represents, from a pharmaceutical perspective, a consistent step forward in trying to standardize a cellular product. This trend in the use of adipose tissue for regenerative purposes may thus facilitate the introduction of clinically translatable protocols, not only for aesthetic purposes but also for other medical indications, such as orthopedics or neurodegenerative diseases [10,11]. This is due to the easier method of isolating ASCs when compared to bone marrow-derived MSCs, which could constitute a potential new vehicle in the development of advanced therapy medicinal products (ATMPs). Few works have been reported on SVF process standardization in the last 10 years [12,13], but the sensitivity of ATMPs used as drugs for regenerative medicine and the number of new candidate drugs containing SVF or only ASCs for therapeutical use are increasing, especially in orthopedics (www.clinicaltrials.gov) [14]. With this purpose, we have published a collaborative work in which we standardize good manufacturing practices (GMP)-compliant isolation and analysis processes in two different and distant clean rooms [15].

Here, we present data from our clean room facility in Switzerland on 302 samples processed following the GMP rules, with the aim of further improving the standardization process of SVF extraction while also considering upstream variability in the operators with attention to adipose tissue, biological patient intra-variability, anatomical site of collection, and, finally, transportation conditions. To our knowledge, these variables have not yet been considered in peer-reviewed papers, even though they might be relevant for this study. The data reported in this study are the outcome of an effort of standardization at the GMP level, considering transport of samples, processing in a clean room, and analysis in terms of cellular composition.

## 2. Materials and Methods

### 2.1. Patients’ Information

All the patients reported in this study were requested to sign an informed consent form, and samples were processed during the period of 2014–2022.

### 2.2. Adipose Tissue Sample Transportation

The transportation of the samples was standardized following GMP guidelines, recognized by the Swiss Regulatory Affairs Body, Swissmedic.

### 2.3. Adipose Tissue Sampling

Liposuction was performed in Switzerland between 2014 and 2022 by 12 recognized plastic surgeons during surgical aesthetic procedures. We included in this study samples from 302 patients: 191 women and 111 men older than 18 years (range 20–77 years), in good health and with negative tests for HIV (human immunodeficiency virus), HCV (hepatitis C virus), and HBV (hepatitis B virus). All patients signed a written informed consent form.

Liposuctions were performed in the operating room under total or local anesthesia, with different cannulas and aspiration devices. Samples of 150 mL of adipose tissue were taken in 3 syringes of 50 mL. The syringes were inserted into sterile bags and transported to a clean room in Switzerland in a GMP-certified transportation box at room temperature (20 ± 10 °C), then processed within 24 h of collection.

### 2.4. GMP Isolation of SVF

Our extraction protocol was based on the use of 100 mL syringes (Omnifix 100 mL with Louer Adaptor, B. Braun AG, Messlungen, Hessen, Germany) as separation funnels [16,17]. The first step was to wash the samples twice with 30 mL of Dulbecco’s phosphate-buffered saline with calcium and magnesium (DPBS +/+, Merck, Darmstadt, Germany), holding the syringe in a vertical position for a few minutes to allow for the separation of the adipose tissue from the aqueous phase, which was discarded. Then, the adipose tissue was enzymatically digested with the same enzyme blend, Liberase or Celase^®^ (Cytori Therapeutics, San Diego, CA, USA), a mixture of proteolytic enzymes, diluted in DPBS +/+ at a final concentration of 0.28 Wünsch U/mL, for 45 min at 37 °C under constant and gentle agitation. The enzymatic reaction was stopped with 30 mL of DPBS without calcium and magnesium (Merck, Darmstadt, Germany), supplemented with 1% human albumin solution. The syringe was returned to a vertical position and the aqueous lower phase, containing the SVF cells, was collected into a conical 50 mL centrifuge tube (Corning Science Mexico SA, Reynosa, Tamaulipas, Mexico). This washing step was performed twice to increase the cell yield. The tubes were centrifuged for 5 min at 400 RCF, and the cellular pellet was resuspended in 10 mL of DPBS −/− with 1% human albumin and filtered using a 100 μm sieve and a 40 μm sieve (Corning Inc., New York, NY, USA). Finally, the hydrophilic phase containing the SVF was centrifuged again at 400 RCF for 5 min RT, and the resulting pellet was resuspended in 5% human albumin (CSL Behring AG, Bern, Switzerland).

#### 2.4.1. SVF Characterization

Before cytofluorimetric characterization, the number of total viable cells (SVF) and the percentage of viability were determined with a Nucleocounter NC-100™ device (Chemometec, Allerod, Denmark). Then, cells were characterized by cytofluorimetric analysis using a 10-channel Navios cytometer (Beckman Coulter Inc., Nyon, Switzerland), and the resulting data were analyzed using Kaluza Software 1.2 (Beckmann Coulter), as previously reported [15]. To ensure the quality of the cytofluorimetric analyses, the exclusion of unspecific signals was achieved with matched isotypic controls. Moreover, fluorescence compensation was performed regularly with the positive/negative method, following the instructions of Beckmann Coulter; for this purpose, a ClearLLab Compensation Kit and VersaComp Antibody Capture beads (Beckman Coulter, T. Nagar Chennai, India) were used. Briefly, 500,000 cells were centrifuged for 5 min at 400 RCF, and the pellet was resuspended in 220 μL of DPBS without Ca^2+^/Mg^2+^ (Merck, Darmstadt, Germany) with 1% human AB serum (Pan-Biotech, Aidenbach, Germany). Subsequently, 100 μL of cell suspension was mixed together with Syto40 (Life Technologies Corporation, Carlsbad, CA, USA) and 7-AAD (Beckmann Coulter Inc., Nyon, Switzerland) in a customized test tube coated with the antibodies CD34, CD146, and CD45 (DuraClone Mix, Beckman Coulter Inc., Nyon, Switzerland). All antibodies were used according to the manufacturer’s instructions. After 20 min of incubation, erythrocytes were lysed with 1 mL of VersaLyse lysing solution (Beckman Coulter Inc., Nyon, Switzerland) for 15 min. After this incubation step, samples were analyzed at the cytometer. Briefly, the DNA marker Syto40 was used to exclude cellular debris (i.e., negative), and 7-AAD was used for dead and live cell discrimination in order to assess the cellular viability [17,18]. ASCs were identified in the CD45 and CD146 negative and CD34 positive fraction [19].

#### 2.4.2. Gating Strategy

**Figure 1:**
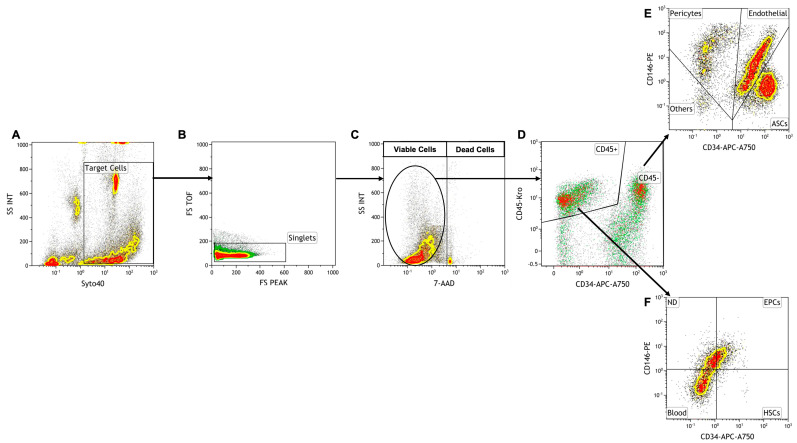
Illustration of the gating strategy. ASCs: adipose-derived stem cells; endothelial: endothelial cells; FS: forward scatter; pericytes: pericytes; SS: side scatter; EPCs: endothelial progenitor cells; HSCs: hematopoietic stem cells; blood: blood cells. (**A**) Target cells; (**B**) single events; (**C**) viable cells (on the left) and dead cells (on the right); (**D**) CD45+ and CD45– cells; (**E**) CD45– cell subpopulations; (**F**) CD45+ cell subpopulations.

Nucleated cells were selected by the nuclear marker Syto40, and, thanks to the forward scatter, cellular aggregates were removed. The presence of the 7-AAD marker discriminated viable cells from dead cells, determining the cellular viability. Among all the viable nucleated cells (VNCs), the staining with the CD45 marker allowed us to discriminate hematopoietic (CD45+) cells from non-hematopoietic (CD45−) cells. On CD45− cells, a density plot (CD146 vs. CD34) was gated to identify ASCs (CD34+, CD146−, CD45−), EPCs (CD34+, CD146+, CD45−), pericytes, and precursor cells (CD34−, CD146+, CD45−). This strategy allowed us to obtain a complete characterization of the SVF (Figure 1).

### 2.5. Statistical Analysis

Statistical analyses were performed using Graph Pad Prism 8.4 (GraphPad Software 8.4 CA, Boston, MA, USA). For N > 2 cohort comparisons, ordinary one-way ANOVA and nonparametric ANOVA were used. For N = 2 cohort comparisons, the unpaired *t*-test and the unpaired nonparametric Mann–Whitney *t*-test were used. A *p*-value ≤ 0.05 was considered to indicate a statistically significant difference.

## 3. Results

Producing ATMPs following GMP guidelines ensures a standardized protocol, normally reviewed and authorized by the local regulatory authority. This guarantees pharmaceutical quality in terms of sterility of the product and number of delivered cells and, finally, a solid tracking system in case of recalls, complaints, or withdrawals. In the last 10 years, we have made significant improvements aimed at standardizing the analysis protocol of cells extracted from the samples arriving at our GMP-compliant facility. Indeed, we recently published a collaborative study with the University of Marseille, grouping 364 patients processed in both GMP facilities, on the harmonization of two slightly different analytical methods to obtain a common protocol for adipose-derived mesenchymal stem cell analysis [15]. This standardization process led us to develop a cytofluorimetric gating strategy to obtain adipose-derived cell subpopulations and characterize them as shown in Figure 1.

SVF cells were extracted from adipose tissue, as previously reported in our paper in 2014 [20], and analyzed through a cytofluorimeter, first selecting nucleated cells with Syto40 (Figure 1A), then removing cellular aggregates (Figure 1B) and discriminating viable cells from total nucleated cells (TNCs) with death marker 7-amino-actinomycin D (7-AAD) (Figure 1C). Then, gated living cells were selected, first for CD45 expression (Figure 1D), discriminating hematopoietic CD45+ cells from CD45− cells, which were successively analyzed for CD34 and CD146 expression (Figure 1E). This gave rise to three different cell subpopulations: ASCs, (Figure 1E, lower right panel), EPCs (Figure 1E, upper right panel) and pericytes (Figure 1E, upper left panel). Other CD45-positive cell subpopulations can be observed in Figure 1F, as previously described [15,16]. This gating strategy was applied to all 302 samples to standardize data production. With this purpose in mind, some of the potential influencing factors were analyzed, including those connected with harvesting operators and anatomical sites of collection, which are sources of variability outside of the researchers’ control. Each surgeon had a preferred collection technique, representing a major hurdle in biodiversity biases and making it difficult to completely standardize the method of adipose tissue collection. We thus grouped the patients based on the surgeon who performed the liposuction, obtaining 12 groups of patients, each with N > 10 patients. For each group, the average number of TNCs per mL of adipose tissue and the average viability were compared to the average of the whole cohort of 302 samples.

**Figure 2:**
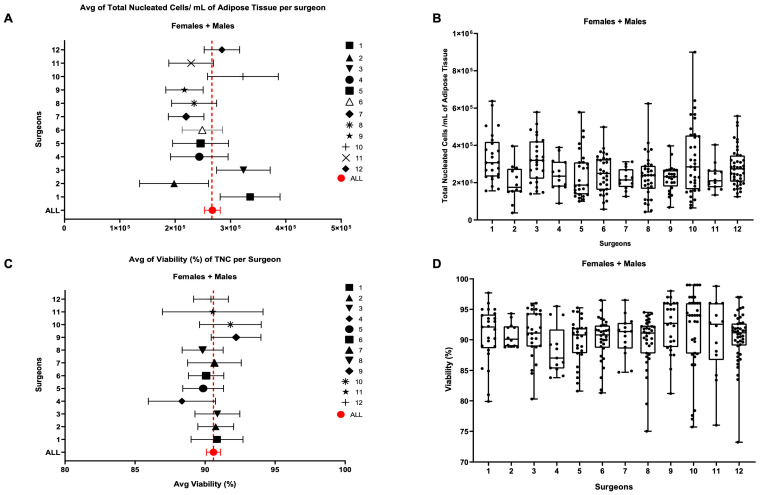
Influence of the surgeon on the number of TNCs per mL of adipose tissue and on the cellular viability. (**A**,**B**) Comparison of the number of TNCs/mL between samples harvested by different surgeons. (**C**,**D**) Cellular viability. Single groups’ average compared with the average across all 302 samples. Females + males cohort, N = 302; females: N = 191, males: N = 111.

Figure 2 shows the results for which no significant statistical differences were found in either parameter between the 12 cohorts of patients treated by different surgeons. This indicates that the surgeon was not a relevant factor influencing TNCs or cell viability. Deeper analysis, which involved splitting every patient cohort by gender, revealed no statistically significant differences (Figure S1), nor did splitting the data based on cell subpopulations such as ASCs, EPCs, and pericytes (Figure S2). The lack of differences between the surgeons led us to check for another possible influencing factor: the anatomical site of collection. For this purpose, we divided the 302 patients into five groups based on the most representative anatomical collection sites: abdomen, flanks, hips, lumbar, and other (which included all the different sites of collection together with unknown and multiple areas). These samples were analyzed against TNCs per mL of adipose tissue and viability.

**Figure 3:**
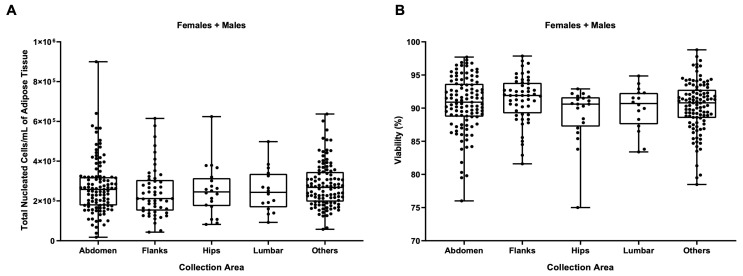
(**A**) Influence of the anatomical area of collection on the number of TNCs per mL of adipose tissue and (**B**) on the cellular viability. Females + males: N = 302, abdomen: N = 107, flank: N = 52, hip: N = 20, lumbar: N = 16, other: N = 107.

As shown in Figure 3, no statistically significant differences were found when comparing the anatomical area of collection, suggesting that this had no impact on the two biological parameters, TNCs per mL of adipose tissue and percentage of viable cells, which are considered the most important parameters for cell therapies. The method used to analyze differences between the surgeons was also used to test differences relating to the collection site: we evaluated TNCs/mL, viability, and all cell types/mL of our characterized subpopulations, splitting each group according to gender. Again, no significant differences were detected (Figures S3 and S4).

**Figure 4:**
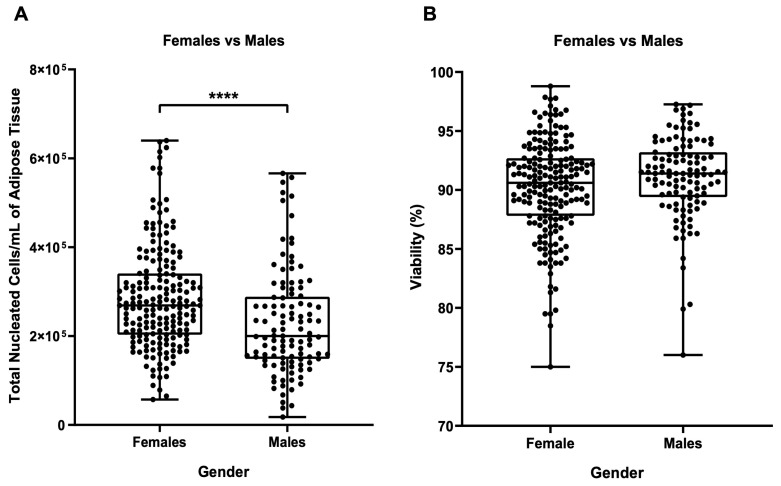
(**A**) Influence of gender on the number of TNCs per mL of adipose tissue. (**B**) Influence of gender on cellular viability. Females + males: N = 302, N = 191 females and N = 111 males. **** *p* ≤ 0.0001.

At this point, it was possible to eliminate two potential influencing factors: the surgeon and the anatomical site of collection, and to proceed to the inter-analysis of two biological parameters, the gender and age of the patient. Surprisingly, there was a significant difference between TNCs per mL of adipose tissue in samples from male and female patients, as shown in Figure 4A (*p* ≤ 0.001), with female samples containing higher numbers of TNCs. However, no statistical differences were detectable between females and males in terms of cell viability (Figure 4B).

**Figure 5:**
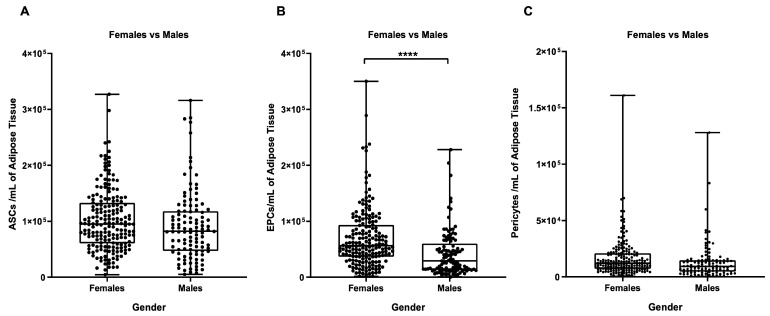
Influence of gender on the number of a specific cell sub-type per ml of adipose tissue. (**A**) ASCs/mL, (**B**) EPCs/mL, and (**C**) pericytes/mL. ****: *p* ≤ 0.0001.

By dividing stromal vascular cells into their main cell subpopulations (ASCs, EPCs, and pericytes) it was possible to more effectively characterize the differences between male and female samples. As shown in Figure 5, the female samples contained more EPCs than the male ones (*p* ≤ 0.0001, Figure 5B).

**Figure 6:**
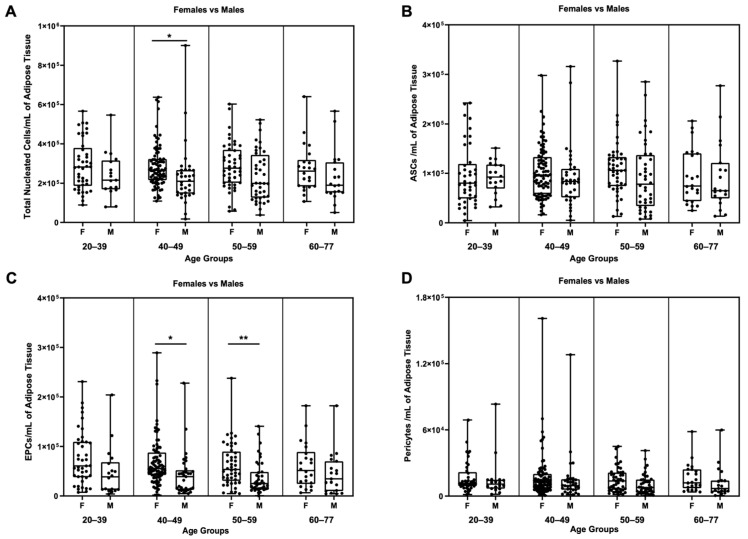
Inter-comparison within the age groups between females and males samples. * *p* ≤ 0.05; ** *p* ≤ 0.005. Comparison of (**A**) TNCs/mL, (**B**) ASCs/mL, (**C**) EPCs/mL, and (**D**) pericytes/mL among females and males in the same age group. N for each group: females (20–39) = 41, males: (20–39) = 18; females (40–49) = 83, males (40–49) = 34; females (50–59) = 45, males (50–59) = 40; females (60–77) = 22, males (60–77) = 19.

Another biological factor, the age of the donors, was then considered. Samples were divided into four age groups: 20–39, 40–49, 50–59, and 60–77. These groups were further split in order to compare the results for male and female samples. There was a statistically significant difference between males and females in the 40–49 age group (Figure 6A, *p* ≤ 0.05) in terms of the number of TNCs per mL of adipose tissue.

A similar difference was detectable in EPCs between males and females in the 40–49 age group (Figure 6C, *p* ≤ 0.05) and the 50–59 age group (Figure 6C, *p* ≤ 0.001).

No statistically significant differences were observed between males and females for ASCs or pericytes within the age-split groups (Figure 6B,D). There were also no significant differences in cellular viability between females and males within the age groupings, nor in the two separated groups (Figure S5).

**Figure 7:**
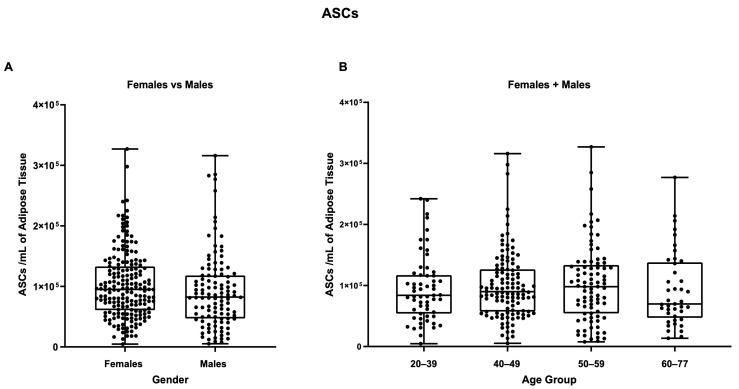
ASCs/mL of adipose tissue. (**A**) Comparison between females and males, and (**B**) between different age groups (females + males cohorts).

The main subpopulations reported in the stromal vascular fraction—that is, ASCs, EPCs, and pericytes—were also compared based on gender and age group. As reported in Figure 7, no statistically significant differences were detectable between male and female samples in the ASCs group, comparing female and male groups with mean values of 1.02 × 10^5^ ASCs per mL and 9.2 × 10^4^ ASCs per ml of adipose tissue, respectively. These results were confirmed by comparing the female + male cohort between the age groups and considering the female and male cohorts separately (Figure S6).

**Figure 8:**
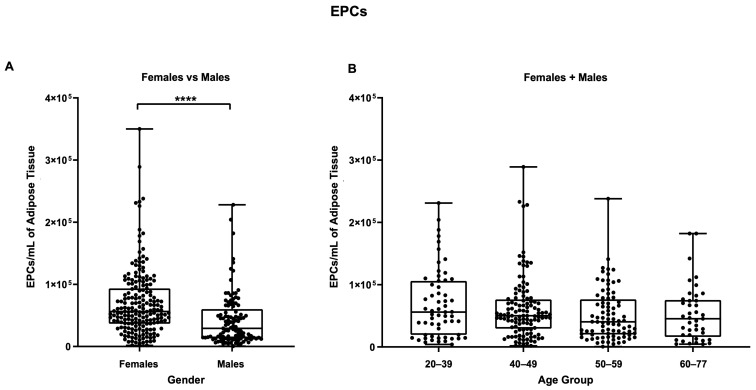
EPCs per mL of adipose tissue. (**A**) Comparison between females and males, (**B**) comparison between different age groups (females + males cohort). **** *p* ≤ 0.0001.

Figure 8 shows the same data analyzed for EPCs, where a statistically significant difference (*p* ≤ 0.0001) was found between the female and male samples. Female samples contained a mean of 6.96 × 10^4^ EPCs per mL, while male samples contained a mean value of 4.26 × 10^4^ cells per mL.

**Figure 9:**
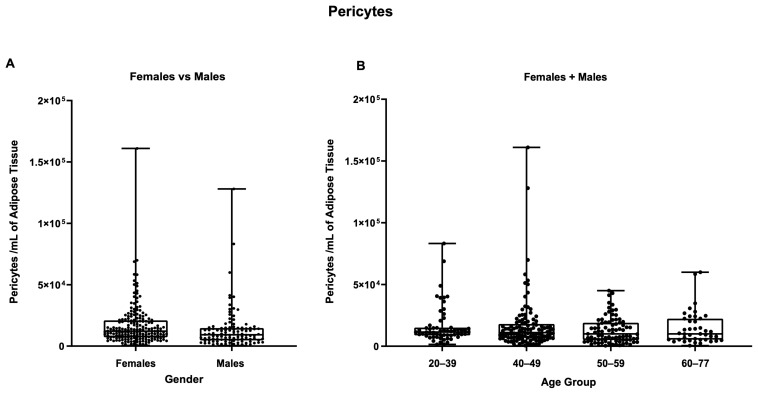
Pericytes/mL of adipose tissue. (**A**) Comparison between females and males, (**B**) comparison between different age groups (females + males cohorts).

Splitting samples by age group did not reveal any further statistically significant differences in either the female + male group or in the separated female and male groups (Figure S7). Finally, as shown in Figure 9, pericytes were present at mean values of 1.66 × 10^4^ per mL of adipose tissue in male samples and 1.31 × 10^4^ pericytes per mL of adipose tissue in female samples. However, the difference was not statistically different, nor were the differences arising from splitting the samples by age group (Figure S8).

Overall, our results suggest that TNC, cell viability, and the distribution of SVF subpopulations are not influenced by the surgeon’s liposuction technique or by the anatomical site of collection. From the intra-sample comparison, the only statistically significant differences that emerged from the 302 samples processed in compliance with GMP procedures were in the concentration of total nucleated cells and, consequently, the number of EPCs per mL in female samples compared to male ones.

The efficacy and safety of aesthetic treatment with SVF was evaluated for 69 patients who received one or two facial injections of autologous SVF resuspended in 5% human albumin solution. After 7 to 38 days, the first check-up was performed by the treating physician, followed by a second one 12 months later. The physician evaluated the skin quality through both visual inspection and pictures.

**Figure 10:**
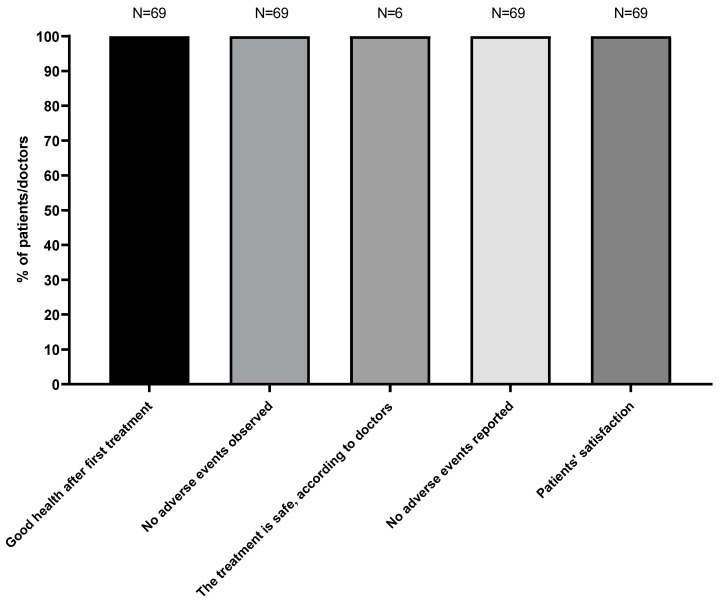
Results of the safety questionnaire completed by N = 69 patients who received a first injection between 2013 and 2020. Column 3 shows that authorized treating doctors all agree that the process was safe.

Patients’ general satisfaction was evaluated via a survey in which they could also report eventual adverse effects. Overall, as shown in Figure 10, 100% of patients were highly satisfied with the treatment, reporting increased skin quality 12 months after the aesthetic procedure. No adverse events were observed by physicians or reported by patients.

## 4. Discussion

The objective of this study was to investigate the biological variability—in terms of number of nucleated cells, viability, and characterized cell subpopulations—of more than 300 samples of adipose tissue processed with a standardized, GMP-compliant protocol in our facilities, with the aim of improving the use of SVF as an ATMP and thus opening the way for its use in cell therapies. The analyzed parameters reflect the quality of the analyzed adipose tissue samples that were the outcome of the production activity. Moreover, these parameters are the most relevant for Switzerland’s national regulatory body (Swissmedic) for cell-based therapies. It was found that, although each surgeon had a preferred technique for adipose tissue harvesting, involving the use of specific cannulas and liposuction devices, this had no significant influence on the biological characteristics of the tissue. This could be due to the fact that all the surgeons participating in our Swiss network are trained in and follow specific guidelines for adipose tissue collection. Similarly, the specific anatomical site from which samples were taken made no significant difference in the results. The results for TNCs/mL and ASCs/mL across the five different anatomical sites of collection were in contrast with previous findings, which reported a higher number of TNCs and ASCs in samples from the inner thigh [21] and a higher yield of ASCs in samples from the abdomen [22]. These contradictory findings may be explained by the different number of samples analyzed (n = 302 versus n = 10, [22]), the different methods used for counting cells (automated vs. manual), and the genders of the donors. The automated cell-counting device reduced the level of inaccurate measurements typical of manual counting, and the high number of samples made the statistical analysis more reliable, even though some differences may have been missed due to the wide variability among the biological samples.

After excluding these two variables, we examined the effects of the genders and the ages of the donors. These two parameters have been investigated primarily in terms of ASCs’ proliferative capacity, differentiation ability, and, more recently, transcriptomic level [23]. In fact, gender dimorphism in adipose tissue in humans has also been widely studied from an anatomical and a physiological angle to examine the differential accumulation of fat depots [24]. This phenomenon is strongly related to hormonal influence [25,26], namely, how estrogen and androgen fluctuation over a lifetime influences the metabolic balance. This gender variability is confirmed by our results: the number of total nucleated cells per mL of tissue was found to be significantly higher among females than males. Upon investigating whether this difference in TNCs was due to a prevalence of a specific cellular subpopulation, it was found that EPCs were consistently higher in females aged 40–59 years than in males of the same age group. This outcome may be explained, as mentioned above, by the larger subcutaneous adipose tissue depots found in females, which require a wider vascular network mainly formed by EPCs interacting with other types of cells. The neovascularization process is stimulated by the action of sex hormones, namely, estrogen and androgen [25,26,27], as they promote the secretion of leptin, a typical adipose tissue-secreted hormone. Leptin stimulates the expression of VEGF by ASCs and endothelial progenitors, which constitute, in concert with pericytes through several molecular interactions, a new vascular network [28,29,30]. EPCs are defined as circulating cells expressing multiple cell surface markers, such as CD34, CD31, vWF (Von-Willebrand Factor), CD146, and CD144 [31,32], and have been demonstrated to provide building blocks for the formation of a new vascular system [33]. As opposed to EPCs cells, when the number of ASCs and pericytes/mL of tissue were compared according to patients’ gender and age, no statistical differences were found. ASCs represent the most interesting population in the field of regenerative medicine, as they are involved in the maintenance of the tissue metabolic balance, angiogenesis, wound healing, and scar reparation [34,35,36,37,38] by also contributing, in synergy with pericytes and EPCs, to sustaining the capillaries of the vascular network [36,39]. Pericytes or perivascular cells retain strong differentiation and angiogenic potential, as recently discovered by Ahmed and colleagues [40], since they display similar characteristics to MSCs and are crucial for ECs’ survival, migration, and growth support [41,42,43]. No other variations in terms of cellular concentrations or cellular viability were found in this study, suggesting that processing samples with a standardized GMP-compliant protocol could better characterize the biological parameters important for further use of these cells as ATMPs, as we have previously shown in a collaborative study [15]. The contradictory findings concerning the number of ASCs/mL based on gender and age-related differences can also be outlined for bone-marrow-derived MSCs (BM-MSCs). Indeed, a recent study by Andrzejewska and colleagues [44] did not find any differences in BM-MSCs preparations among females and males in terms of differentiation or proliferation, contrary to findings previously reported by Siegel and colleagues [45]. In fact, they found smaller MSCs with a high rate of proliferation and more rapid division in female-derived BM-MSCs [45].

This characterization, together with the data on the efficacy and safety of the treatment, could help in defining new cellular standards for ATMPs’ use in cell therapy, as we established GMP-prepared stromal vascular fraction cell release criteria with defined cell parameters.

## 5. Conclusions

Overall, our analyses did not reveal any significant differences resulting from the diversity of surgeons’ techniques or the anatomical site of collection. However, it did outline some gender- and age-related variations. Females aged 40–59 years were found to have a higher concentration of TNCs/mL, and, consequently, of EPCs/mL, compared to males in the same age group. This gender-specific variation may be explained by the different levels of hormones which lead to different accumulations of depots of subcutaneous fat. There was no significant difference in the concentration of ASCs/mL depending on the anatomical area of collection or when comparing gender and age groups. This outcome is in contrast with previous findings, which pointed to a higher number of ASCs in specific areas of the body and a decrease in ASC concentration as the age of the donor increases. The observed results may be due to the significant variability among our biological samples. In conclusion, we can affirm that processing samples in a GMP-compliant environment and with a GMP-compliant protocol could help to define new cellular standards for ATMPs’ use as pharmaceutical drugs in cell therapy.

## Data Availability

All raw data are available upon request.

## Supplementary Materials

The following supporting information can be downloaded at: https://www.mdpi.com/article/10.3390/biomedicines11092533/s1, Figure S1: Comparison of the number of TNCs/mL of adipose tissue and cell viability in separate females and males cohorts based on surgeons. Figure S2: Subpopulations analyses in females and males cohorts. Figure S3: Comparison of the number of TNCs/mL of adipose tissue and viability between the different anatomical areas of collection in females and males. Figure S4: Subpopulation analyses in females and males cohorts, comparing different anatomical areas of collection. Figure S5: viability comparison between four age groups in both the females + males cohort and separate females and males cohorts. Figure S6: Comparison of the number of ASCs/mL of adipose tissue in the females + males cohort and separate females and males cohorts between age groups. Figure S7: Comparison of the number of EPCs/mL of adipose tissue in the females + males cohort and separate females and males cohorts between age groups. Figure S8: Comparison of the number of pericytes/mL of adipose tissue in the females + males cohort and separate females and males cohorts between age groups.
