# Supplementary material for: Processing Adipose Tissue Samples in a GMP Environment Standardizes the Use of SVF in Cell Therapy Treatments: Data on 302 Patients
Source: Biomedicines. 2023 Sep 14;11(9):2533. doi: 10.3390/biomedicines11092533 (PMC10525825; doi:10.3390/biomedicines11092533)
Supplement: Supplementary file 1 [file biomedicines-11-02533-s001.zip › biomedicines-2526551-supplementary.pdf]

Supplementary Materials

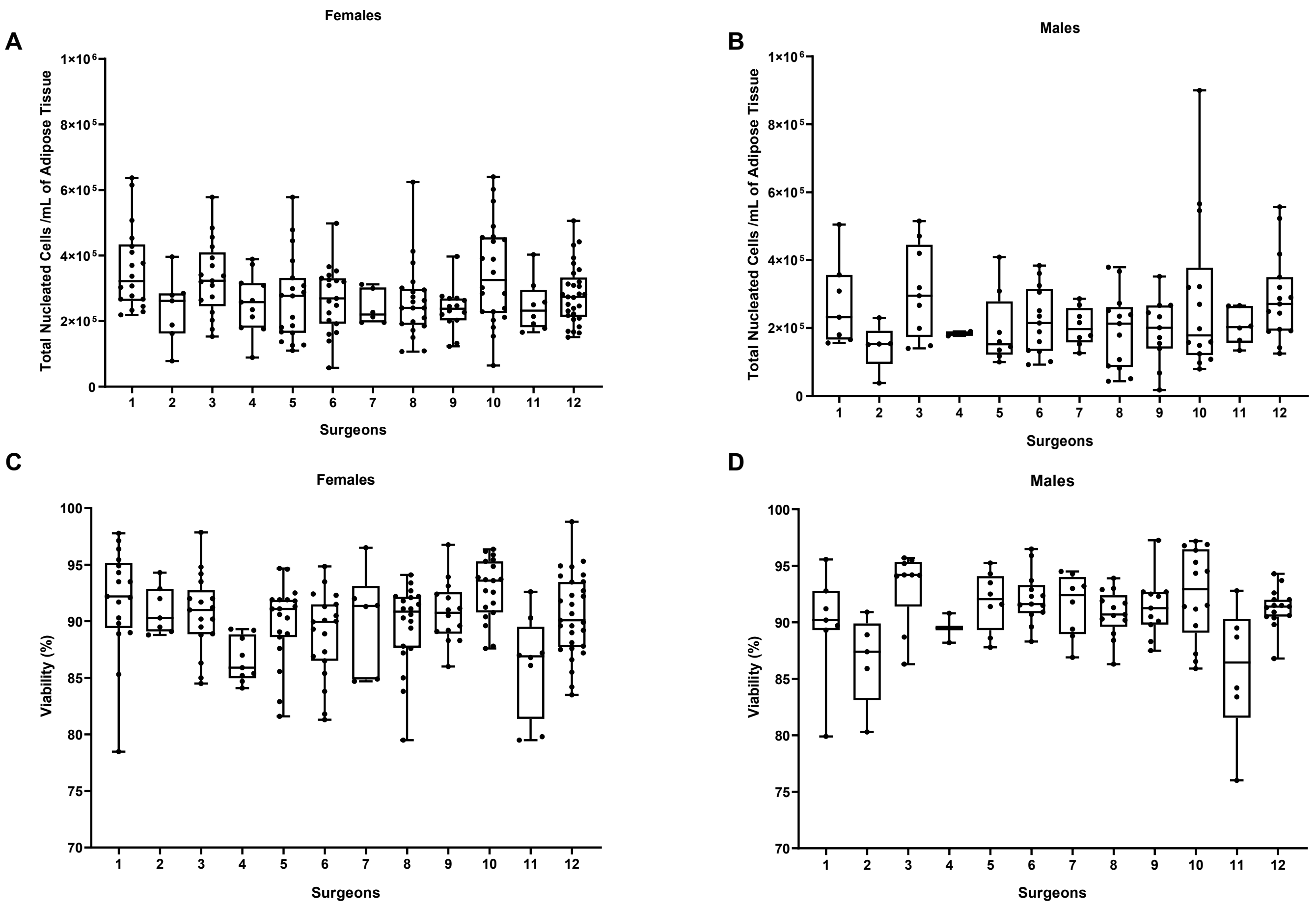

Figure S1. *Comparison of the number of TNCs/mL of adipose tissue* ***(A,B)*** *and cellular viability* ***(C,D)*** *in separately females and males cohorts based on surgeons.*

Females: 1. N=18, 2. N=7, 3. N=17, 4. N=11, 5. N=19, 6. N=19, 7. N=6, 8. N=21, 9. N=14, 10. N= 22, 11. N=8, 12. N=29.

Males: 1. N=7, 2. N=5, 3. N=9, 4. N=2, 5. N=8, 6. N=13, 7. N=8, 8. N=13, 9. N=11, 10. N= 14, 11. N=6, 12. N=15.

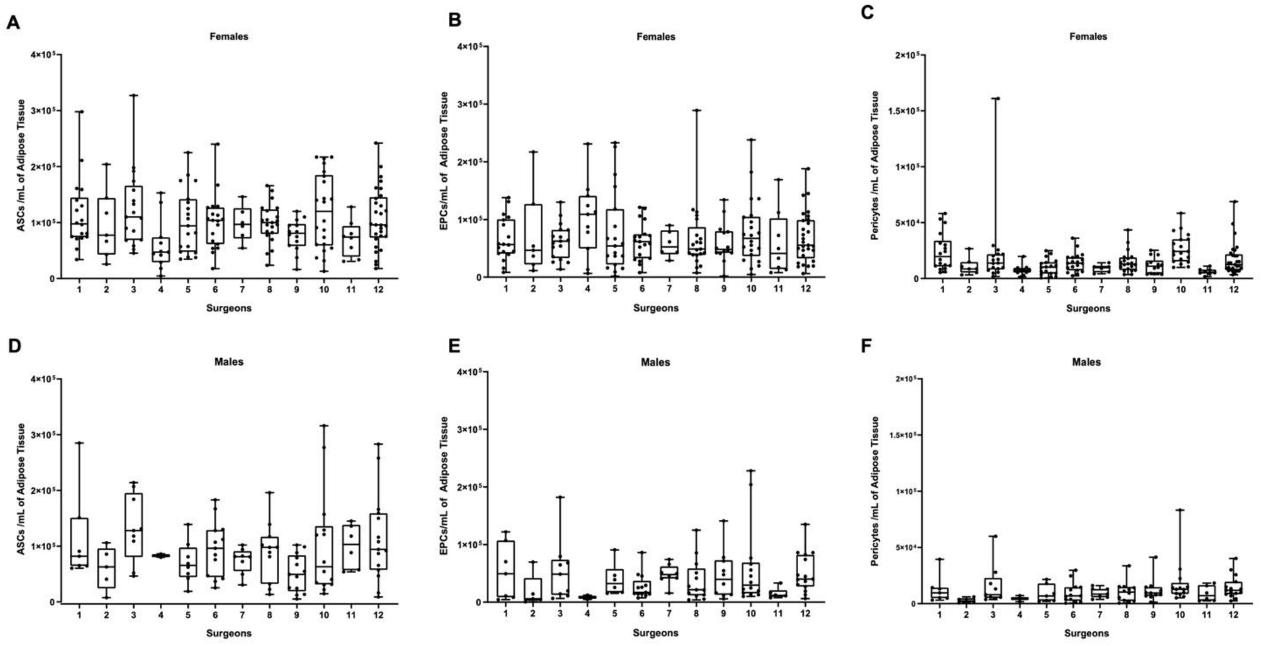

Figure S2. *Sub–populations analyses in females and males cohorts.*
(A,B,C) Females: 1. N=18, 2. N=7, 3. N=17, 4. N=11, 5. N=19, 6. N=19, 7. N=6, 8. N=21, 9. N=14, 10. N= 22, 11. N=8, 12. N=29.
(D,E,F) Males: 1. N=7, 2. N=5, 3. N=9, 4. N=2, 5. N=8, 6. N=13, 7. N=8, 8. N=13, 9. N=11, 10. N= 14, 11. N=6, 12. N=15.

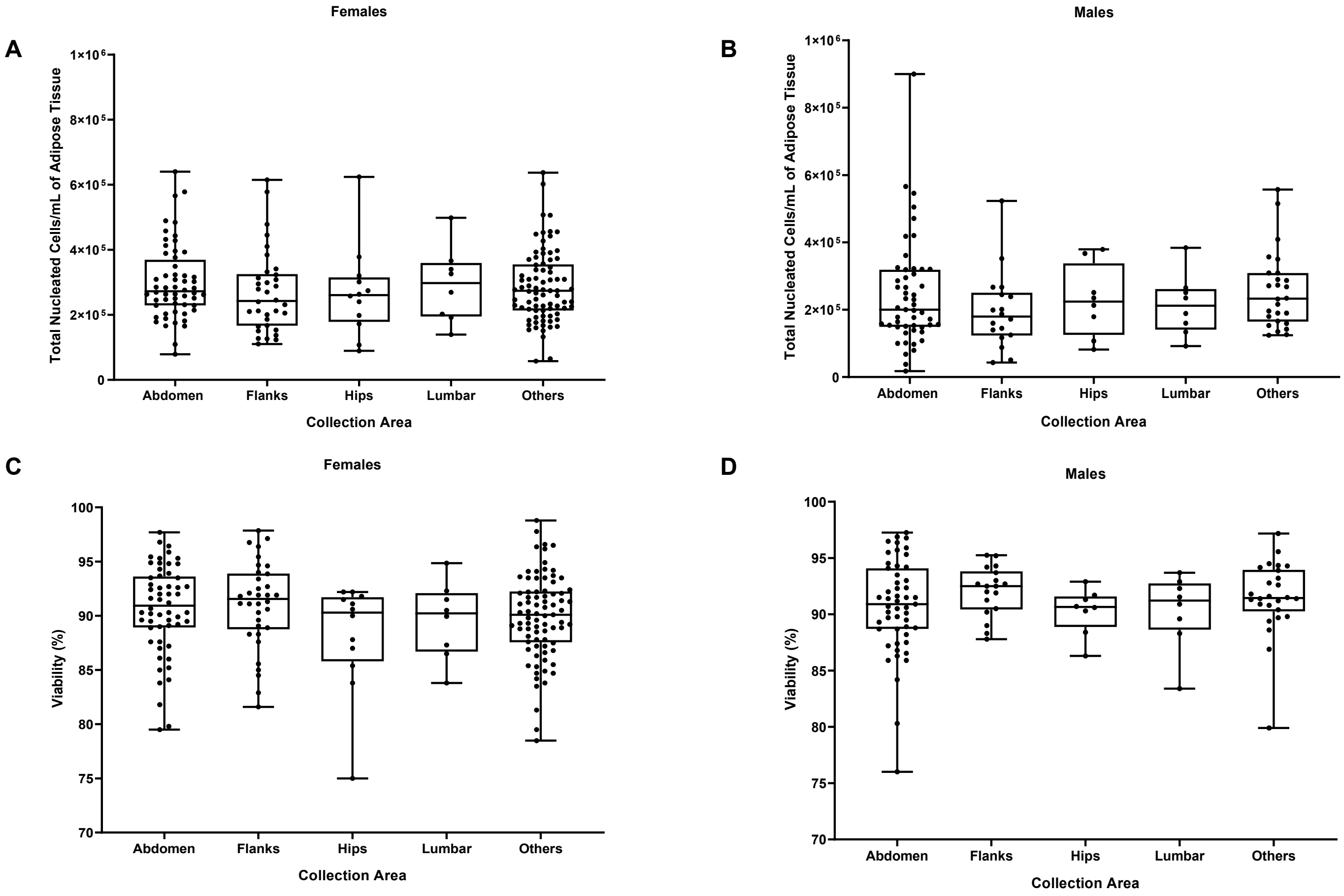

Figure S3. *Comparison of the number of TNCs/mL of adipose tissue* ***(A,B)*** *and viability* ***(C,D)*** *between the different anatomical areas of collection in females and males.*

In females: Abdomen N=56, flanks N=34, Hips N=12, lumbar N=8, others N=81.

In males: Abdomen N=51, flanks N=18, hips N=8, lumbar N=8, others N=26

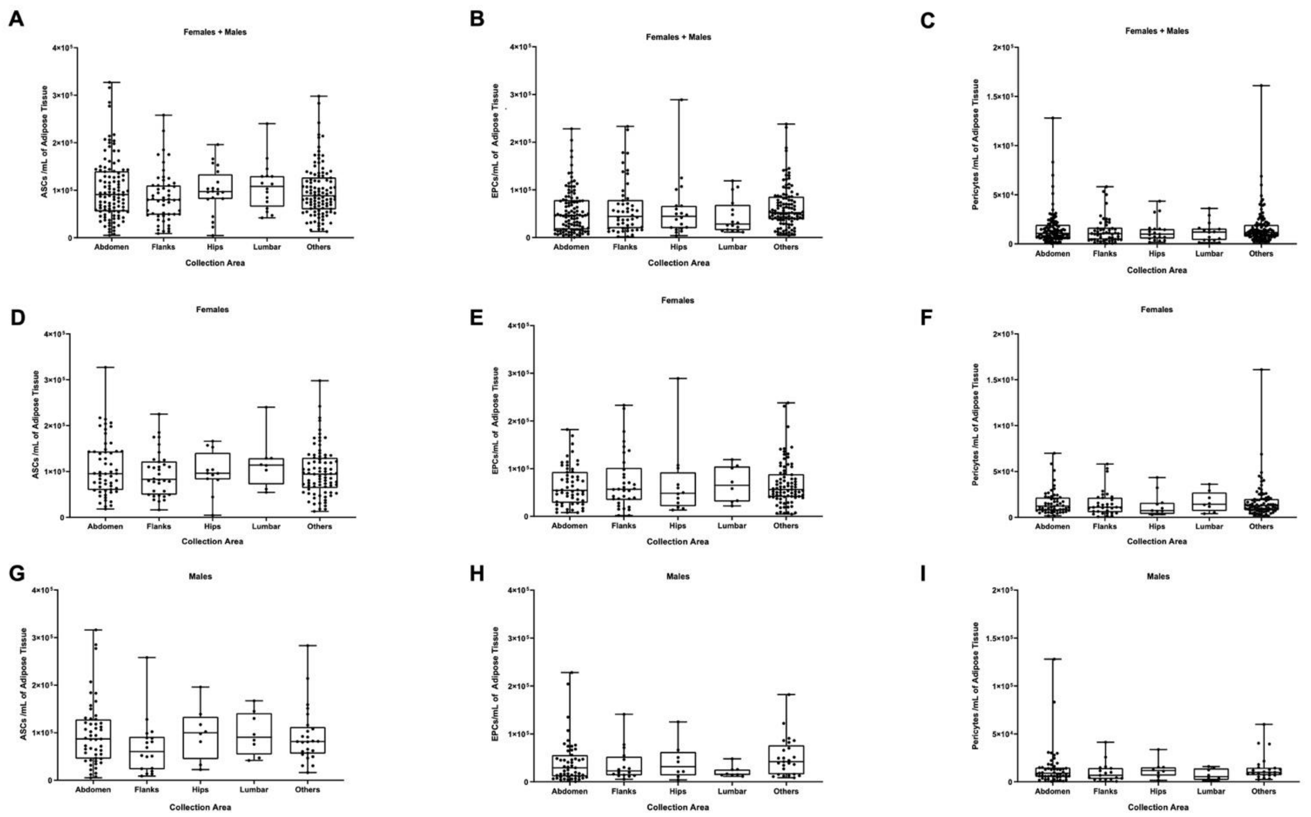

Figure S4. *Subpopulations analyses in females and males cohorts, comparing different anatomical areas of collection.*

(A, B, C) Females + males N=302.

(D, E, F) Females: Abdomen N=56, flanks N=34, hips N=12, lumbar N=8, others N=81.

(G, H, I) Males: Abdomen N=51, flanks N=18, hips N=8, lumbar N=8, others N=26.

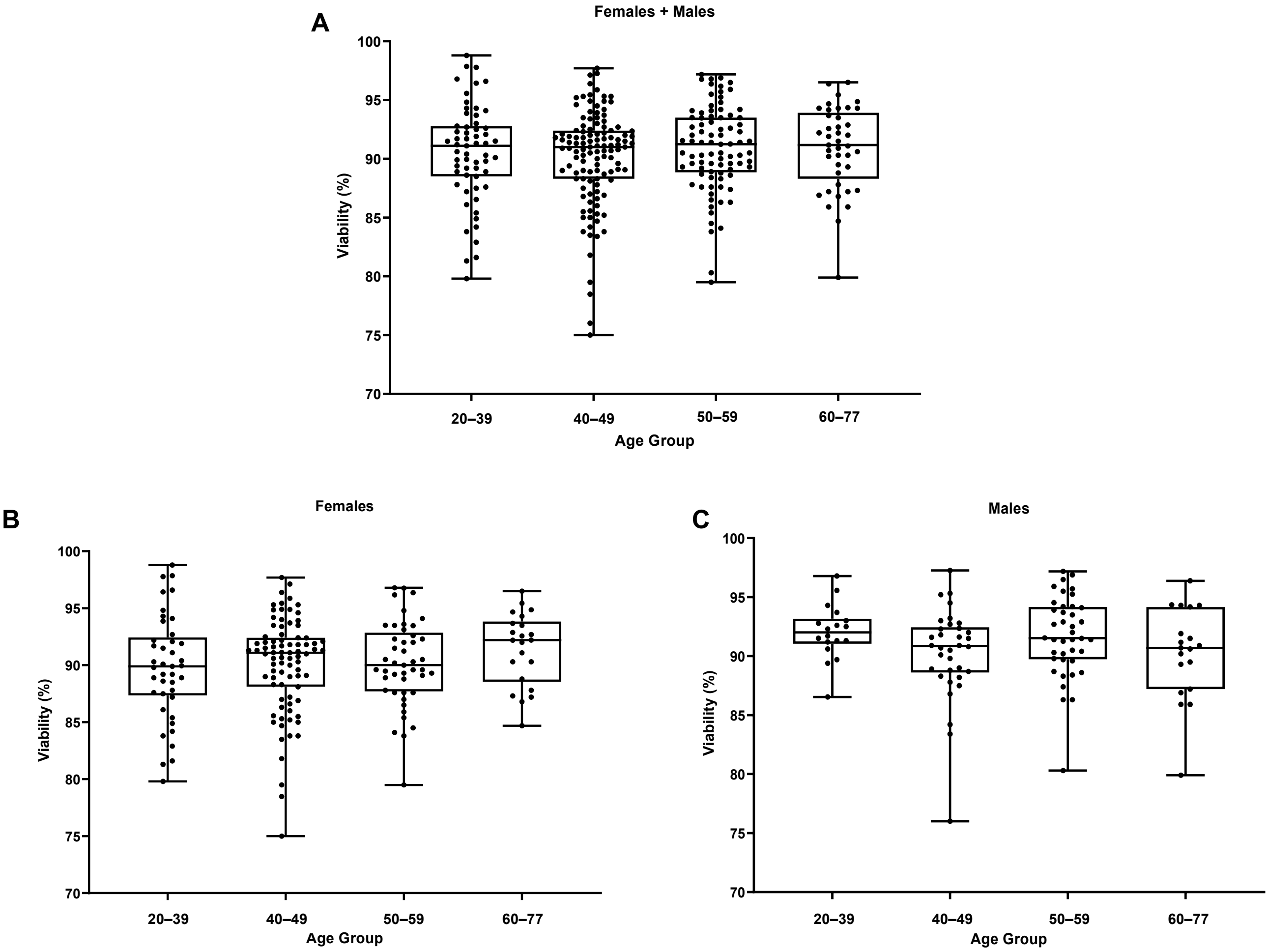

Figure S5. *Viability comparison between four age groups in both the females + males cohort and separate females and males cohorts.*

(A) Females + males: (20–39) N=59, (40–49) N=117, (50–59) N=85, (60–77) N=41.

(B) Females: (20–39) N=41, (40–49) N=83, (50–59) N=45, (60–77) N=22.

(C) Males: (20–39) N=18, (40–49) N=34, (50–59) N=40, (60–77) N=19.

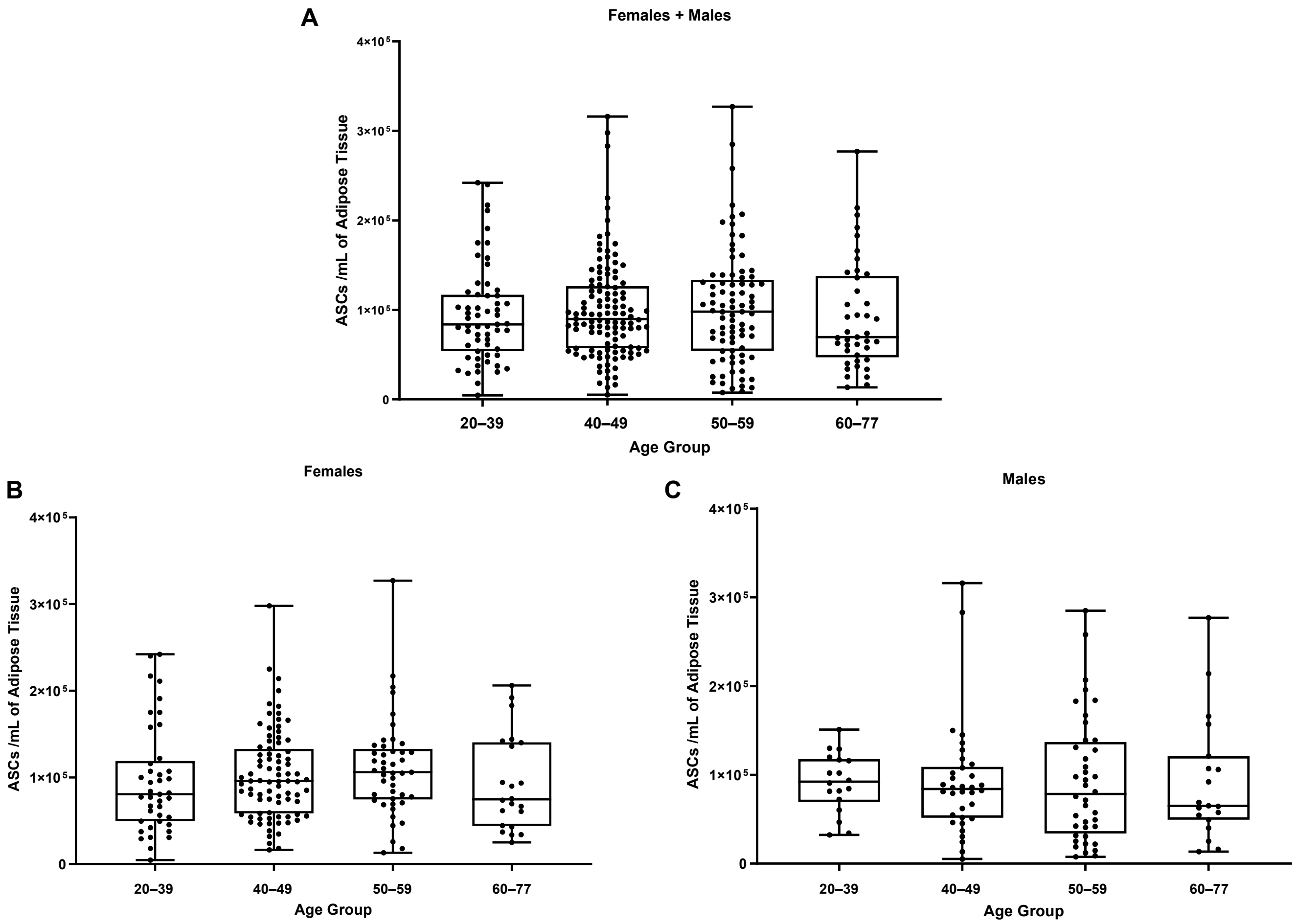

Figure S6. *Comparison of the number of ASCs/mL of adipose tissue in the females + males cohort and separate females and males cohorts between age groups.*

(A) Females+ males: (20–39) N=59, (40–49) N=117, (50–59) N=85, (60–77) N=41.

(B) Females: (20–39) N=41, (40–49) N=83, (50–59) N=45, (60–77) N=22.

(C) Males: (20–39) N=18, (40–49) N=34, (50–59) N=40, (60–77) N=19

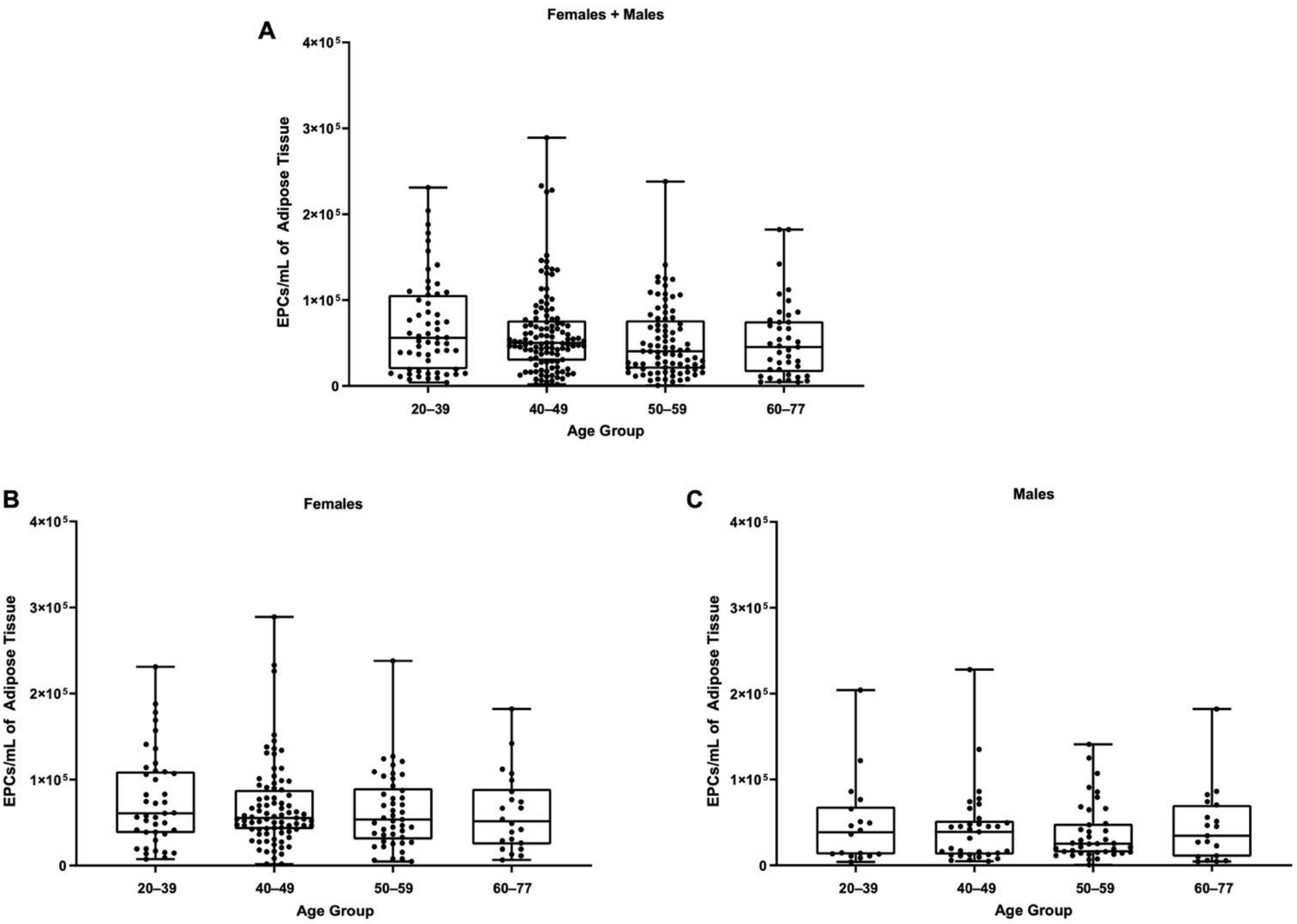

Figure S7. *Comparison of the number of EPCs/mL of adipose tissue in the females + males cohort and separate females and males cohorts between age groups.*

(A) Females+ Males: (20–39) N=59, (40–49) N=117, (50–59) N=85, (60–77) N=41.

(B) Females: (20–39) N=41, (40–49) N=83, (50–59) N=45, (60–77) N=22.

(C) Males: (20–39) N=18, (40–49) N=34, (50–59) N=40, (60–77) N=19.

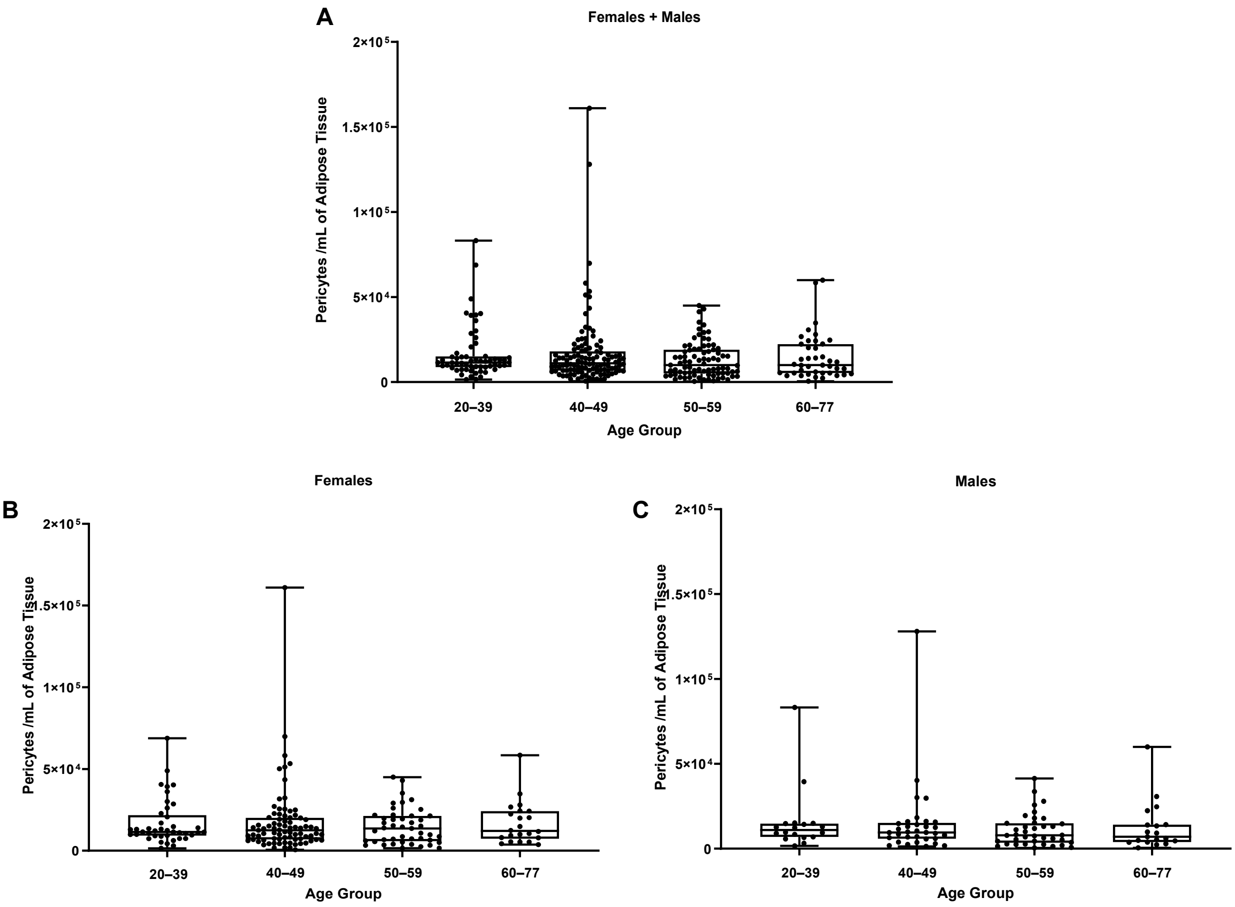

Figure S8. *Comparison of the number of pericytes/mL of adipose tissue in the females + males cohort and separate females and males cohorts between age groups.*

(A) Females + males: (20–39) N=59, (40–49) N=117, (50–59) N=85, (60–77) N=41.

(B) Females: (20–39) N=41, (40–49) N=83, (50–59) N=45, (60–77) N=22.

(C) Males: (20–39) N=18, (40–49) N=34, (50–59) N=40, (60–77) N=19.
